# Generative chromoendoscopy for gastric neoplasms: a multicenter prospective study

**DOI:** 10.1055/a-2873-4466

**Published:** 2026-06-11

**Authors:** Sho Suzuki, Yusuke Monno, Hiroyuki Yamamoto, Ayaka Takasu, Toshiaki Hirasawa, Toshihiro Nishizawa, Shun Ito, Fumiaki Ishibashi, Masatoshi Okutomi, Tomohiro Tada

**Affiliations:** 1 Department of Gastroenterology 38259 International University of Health and Welfare Ichikawa HospitalIchikawaChibaJapan; 2 Faculty of Medicine, Graduate School of MedicineInternational University of Health and WelfareNaritaJapan; 3 Department of Systems and Control Engineering, School of Engineering 13290 Institute of Science TokyoTokyoJapan; 4 Department of GastroenterologyCancer Institute Hospital, Japanese Foundation for Cancer ResearchTokyoJapan; 5 Department of Gastroenterology 625200 International University of Health and Welfare Narita HospitalNaritaJapan; 6 AI Medical Service Inc.TokyoJapan; 7 J’s Clinic of Gastrointestinal Endoscopy & ProctologySaitamaJapan

## Abstract

**Background:**

Generative indigo carmine chromoendoscopy (generative chromoendoscopy), produced using deep learning-based synthetic image transformation, is a novel image-enhanced endoscopic approach, whose clinical feasibility has not been validated. This proof-of-concept study evaluated whether generative chromoendoscopy improves the visibility of gastric neoplasms compared with conventional white-light imaging (WLI) and real chromoendoscopy.

**Methods:**

This prospective multicenter study was conducted at three Japanese institutions between January and August 2025. A deep neural network-based program converted WLI into generative chromoendoscopy images. Patients with early gastric cancer or adenoma sequentially underwent endoscopy using WLI, generative chromoendoscopy, and real chromoendoscopy. During live endoscopy, two endoscopists independently assessed lesion visibility using a 7-point Likert-type scale (−3 to +3). The primary end point was visibility of gastric neoplasms with generative chromoendoscopy relative to WLI, with comparison to real chromoendoscopy. Secondary end points included clinicopathologic factors associated with improved visibility.

**Results:**

60 patients were included. Mean (SD) visibility scores were 0.67 (0.93) for generative chromoendoscopy and 0.51 (1.00) for real chromoendoscopy. The mean difference was 0.16 (95%CI 0.01 to 0.30). Differentiated-type histology (odds ratio [OR] 2.19, 95%CI 1.03 to 4.62), current *Helicobacter pylori* infection (OR 2.14, 95%CI 1.02 to 4.46), and expert endoscopist status (OR 2.14, 95%CI 1.22 to 3.76) were significantly associated with higher visibility scores using generative chromoendoscopy.

**Conclusions:**

Generative chromoendoscopy was feasible during live endoscopic procedures. It improved visibility of pre-identified gastric neoplasms compared with WLI and achieved visibility comparable to real chromoendoscopy.

## Graphical Abstract

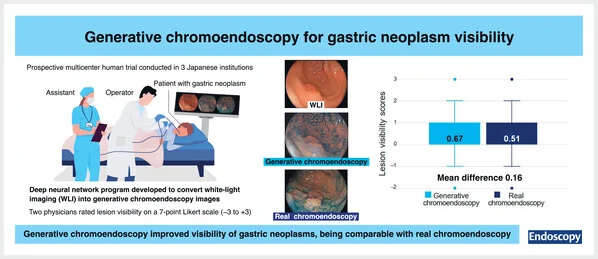

## Introduction

Gastric cancer is the fifth most common malignancy worldwide and accounts for approximately 660000 deaths annually 1 . Early detection and treatment markedly improve prognosis and survival. In countries with a high prevalence of gastric cancer, national screening programs have been implemented to facilitate early diagnosis and reduce mortality 2 3 4 . Among available modalities, endoscopy remains the most reliable screening method 5 . Nevertheless, early gastric cancer can be difficult to detect endoscopically because of its subtle morphologic and color changes. In addition, diagnostic performance varies considerably depending on factors such as endoscopist experience and equipment quality.

Indigo carmine chromoendoscopy is a conventional technique that enhances visualization of gastrointestinal epithelial lesions by accentuating mucosal irregularities and color contrast. Observational studies have suggested that chromoendoscopy may aid in the diagnosis of early gastric cancer; however, its use is limited by additional costs and procedural complexity, including dye preparation and spraying, which have hindered widespread adoption.

Deep neural networks, a subset of machine learning, have recently gained prominence in medical imaging. The cycle-consistent generative adversarial network (CycleGAN) is a deep learning architecture designed for image-to-image translation 6 . CycleGAN learns mappings between two image domains using unpaired datasets, eliminating the need for paired training samples 7 . We developed a CycleGAN-based program capable of converting white-light imaging (WLI) into generative chromoendoscopy images of gastric neoplasms 8 . In our previous study, generative chromoendoscopy showed potential for improvement in the diagnosis of gastric neoplasms; however, that analysis was limited to retrospective evaluation of still images.

To address this gap, we further refined the generative chromoendoscopy system to achieve real-time imaging as a seamless video stream during endoscopy, enabling its use as a standalone image-enhanced endoscopy (IEE) modality in clinical practice. This proof-of-concept study reports the first clinical application of generative chromoendoscopy using deep learning-based synthetic image transformation in humans and aims to evaluate its effectiveness in improving the visibility of gastric neoplasms compared with both conventional WLI and real chromoendoscopy.

## Methods

### Study design and ethics

This prospective multicenter study was conducted in accordance with the principles of the Declaration of Helsinki. The study protocol was approved by the Certification of Clinical Trials Review Board of Nihon University Itabashi Hospital on 9 October 2024 (approval no. CR2410–018), which served as the central review board for all participating sites. Written informed consent was obtained from all participants prior to their enrolment.

### Development of the generative chromoendoscopy system

Details of system development have been reported previously 8 9 . In this study, we used the same system as in our prior research 10 . A brief description is provided here.

Endoscopic images used for the development of generative chromoendoscopy were obtained using standard and high definition endoscopes manufactured by Olympus Corporation (Tokyo, Japan) and Fujifilm Corporation (Tokyo, Japan). The generative chromoendoscopy system was developed using a CycleGAN trained on 2089 WLI images and 2007 real chromoendoscopy images of gastric neoplasms (722 from CV260/290 systems and 1285 from VP7000 systems) collected from 262 patients.

As reported previously 8 9 , CycleGAN was applied for unpaired image-to-image translation to create generative chromoendoscopy images from WLI. To account for manufacturer-specific imaging characteristics, separate CycleGAN models were trained for the Olympus CV260/290 and Fujifilm VP7000 datasets, using identical architectures and hyperparameters.

The trained CycleGAN program was implemented on a laptop equipped with both a central processing unit (CPU) and a graphics processing unit (GPU). Endoscopy systems (CV290 and CV1500, Olympus Corporation; VP7000, and EP8000, Fujifilm Corporation) were connected to the laptop via a digital visual interface or serial digital interface. WLI input was converted in real time into generative chromoendoscopy images and displayed on a secondary monitor at resolutions of 1920 × 1080 or 3840 × 2160.

### Patients and setting

Patients were enrolled at three Japanese institutions between January and August 2025. Eligible participants were consecutive patients aged 20–89 years scheduled to undergo endoscopic submucosal dissection (ESD) for gastric neoplasms. Indications for ESD followed the absolute or expanded criteria specified in the 2021 Japanese Gastric Cancer Treatment Guidelines (6th edition) 11 . Histologic subtypes included papillary adenocarcinoma (pap), well-differentiated tubular adenocarcinoma (tub1), moderately differentiated tubular adenocarcinoma (tub2), poorly differentiated adenocarcinoma (por), signet-ring cell carcinoma (sig), special types (e.g. gastric adenocarcinoma of fundic-gland type), and adenoma, as defined by the Japanese classification of gastric carcinoma 12 . Exclusion criteria were prior gastric surgery, severe co-morbidities, pregnancy or breastfeeding, and inability or refusal to provide informed consent.

### Procedure and outcomes

During the scheduled ESD procedure, while under intravenous sedation, patients underwent endoscopic assessment using the generative chromoendoscopy system. Target gastric lesions were sequentially examined with WLI and generative chromoendoscopy at three distances (distant, medium, and close views). Lesions were then observed using real chromoendoscopy, in which a 0.1% indigo carmine dye solution (Alfresa Pharma Corporation, Osaka, Japan) was sprayed onto the mucosa. Each image set (WLI, generative chromoendoscopy, and real chromoendoscopy) was displayed either sequentially on a single monitor or on two monitors positioned at a distance from each other. Evaluators did not view images from multiple modalities simultaneously and did not cross-reference them during evaluation. Two endoscopists (the operator and an assistant) independently evaluated all images and recorded their scores on separate sheets without verbalizing them. In cases in which the operator could not interrupt the procedure to record scores, the assistant first completed the evaluation, after which the operator verbally conveyed the score to a third-party physician for documentation.

The primary end point was the visibility of gastric neoplasms with generative chromoendoscopy compared with WLI. Visibility was assessed using the pair-comparison method, based on recommendations of the International Telecommunication Union Telecommunication Standardization Sector/Radiocommunication Sector (ITU-T/R) for subjective video quality assessment 13 14 . The pair-comparison method uses a 7-point scale to rate visibility of generative or real chromoendoscopy relative to WLI: +3, much better than WLI; +2, better than WLI; +1, slightly better than WLI; 0, about the same; −1, slightly worse than WLI; −2, worse than WLI; and −3, much worse than WLI ( Table 1 ). This definition is similar to other endoscopic visibility scales. Real chromoendoscopy visibility was also assessed using this scale.

**Table:** **Table 1** Pair-comparison scale for assessing visibility of gastric neoplasms.

Score	Definition
+3	Chromoendoscopy visibility is much better than WLI
+2	Chromoendoscopy visibility is better than WLI
+1	Chromoendoscopy visibility is slightly better than WLI
0	Chromoendoscopy visibility is about the same as WLI
−1	Chromoendoscopy visibility is slightly worse than WLI
−2	Chromoendoscopy visibility is worse than WLI
−3	Chromoendoscopy visibility is much worse than WLI
WLI, white-light imaging.	

Secondary end points were factors influencing lesion visibility with generative chromoendoscopy. These included lesion characteristics (macroscopic type, histologic type, and depth of invasion), degree of gastric mucosal atrophy (Kimura-Takemoto classification) 15 , presence of intestinal metaplasia on histology, *Helicobacter pylori* infection status, type of endoscopic system used, and endoscopist expertise. Endoscopists were classified as experts if they were board-certified trainers of the Japan Gastroenterological Endoscopy Society (JGES); all others were classified as nonexperts.

### Sample size calculation and statistical analyses

In a preliminary investigation, the mean (SD) visibility score of real chromoendoscopy relative to WLI for gastric neoplasms was 0.9 (1.2). In this study, the expected mean and SD for generative chromoendoscopy were 1.0 and 1.0, respectively, indicating visibility comparable to or greater than that of real chromoendoscopy. Based on these assumptions, a sample size of 58 patients was calculated to provide 80% power at a two-sided α level of 0.05 using a *t* test. To account for an anticipated 5% dropout rate owing to ineligible patients or potential system malfunction, the target enrollment was set at 60 patients. A clustering adjustment was not included in the initial sample size calculation.

Categorical variables are presented as number and percentage, and continuous variables as mean (SD). Factors associated with visibility using generative chromoendoscopy were evaluated with a generalized linear mixed model, with “facility” included as a random intercept. Intraclass correlation coefficients (ICCs) were calculated using linear mixed models. All *P* values were two-sided, and values <0.05 were considered statistically significant. Statistical analyses were performed using JMP Student Edition version 18.2.1 (JMP Statistical Discovery LLC, Cary, North Carolina, USA) and R version 4.4.3 (R Foundation for Statistical Computing, Vienna, Austria).

## Results

### Patient and lesion baseline characteristics

During the study period, 64 patients scheduled for ESD were enrolled. One patient was excluded because of a system usage error, and three were excluded because their final pathologic diagnoses were not gastric neoplasms. Therefore, 60 patients (60 lesions) assessed by 19 endoscopists using WLI, generative chromoendoscopy, and real chromoendoscopy were included in the analysis.

Baseline demographic, lesion, and procedural characteristics are summarized in Table 2 . The mean (SD) age of the patients was 69.7 (10.5) years, and 39 (65.0%) were men. A total of 19 endoscopists participated in the visibility assessments, comprising five experts (26.3%) and 14 nonexperts (73.7%). Lesions were most frequently located in the lower third of the stomach (n = 35 [58.3%]), followed by the middle third (n = 13 [21.7%]) and upper third (n = 12 [20.0%]). The most common macroscopic type was depressed (0–IIc; n = 30 [50.0%]), followed by elevated (0–IIa or 0–I; n = 25 [41.7%]) and flat (0–IIb; n = 5 [8.3%]). The mean (SD) lesion size was 12.8 (7.6) mm. Histologically, 42 lesions (70.0%) were differentiated-type adenocarcinomas (tub1 or tub2), eight (13.3%) were adenomas, seven (11.7%) were undifferentiated-type adenocarcinomas (por or sig), and three (5.0%) were adenocarcinomas of the fundic-gland type. The depth of invasion was T1a in 40 patients (66.7%) and T1b in 12 (20.0%).

**Table:** **Table 2** Baseline characteristics of the 60 included patients and their gastric neoplasms (n = 60).

Characteristic	n (%), unless otherwise stated
Patient	
Age, mean (SD), years	69.7 (10.5)
Sex, men	39 (65.0%)
*H. pylori* status	
Negative	12 (20.0%)
Current infection	12 (20.0%)
Eradicated	36 (60.0%)
Lesion	
Location	
Upper	12 (20.0%)
Middle	13 (21.7%)
Lower	35 (58.3%)
Morphology	
Elevated (0–IIa/0–I)	25 (41.7%)
Flat (0–IIb)	5 (8.3%)
Depressed (0–IIc)	30 (50.0%)
Size, mean (SD), mm	12.8 (7.6)
Histologic type	
Adenoma	8 (13.3%)
Differentiated type	42 (70.0%)
Undifferentiated type	7 (11.7%)
Adenocarcinoma of fundic-gland type	3 (5.0%)
Invasive depth	
Adenoma	8 (13.3%)
T1a	40 (66.7%)
T1b	12 (20.0%)
Endoscopic gastric atrophy	
C-0	10 (16.7%)
C1–3	10 (16.7%)
O1–3	40 (66.7%)
Histologic intestinal metaplasia, present	44 (73.3%)
Procedure	
Operator endoscopist level, expert	37 (61.7%)
Assistant endoscopist level, expert	17 (28.3%)
Endoscopy system	
CV290	2 (3.3%)
CV1500	35 (58.3%)
VP7000	11 (18.3%)
EP8000	12 (20.0%)

### Visibility of gastric lesions using generative and real chromoendoscopy

A representative video showing a 10-mm type 0–IIc early gastric cancer on white-light imaging (WLI), generative chromoendoscopy, and real chromoendoscopy.Video 1

**Fig. 1:**
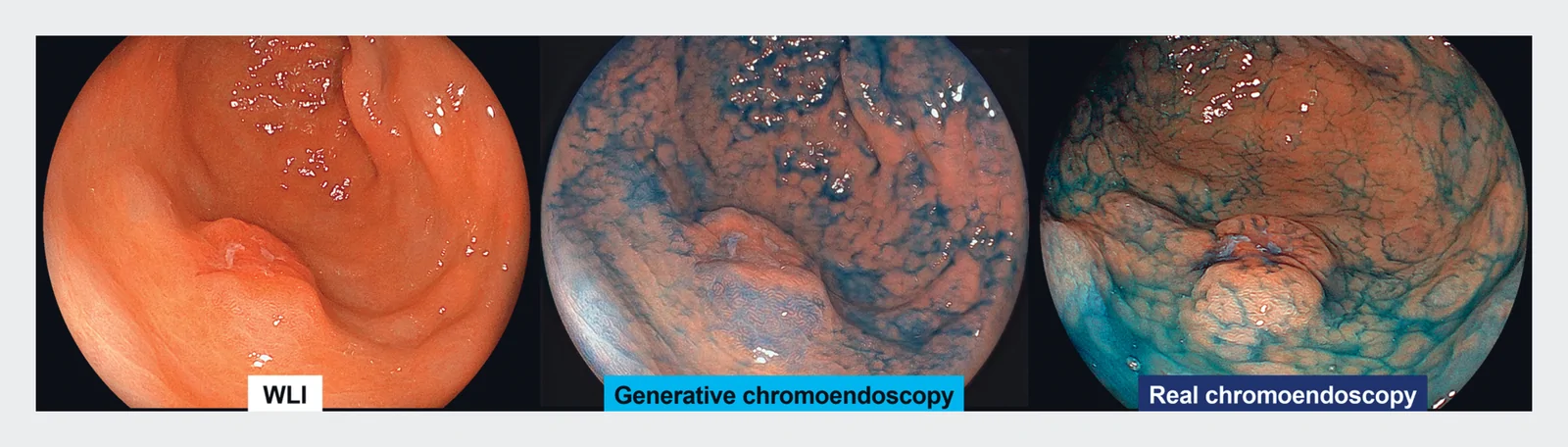
Representative endoscopic images of a type 0–IIa + IIc early gastric cancer obtained with white-light imaging (WLI), generative chromoendoscopy, and real chromoendoscopy.

**Fig. 2:**
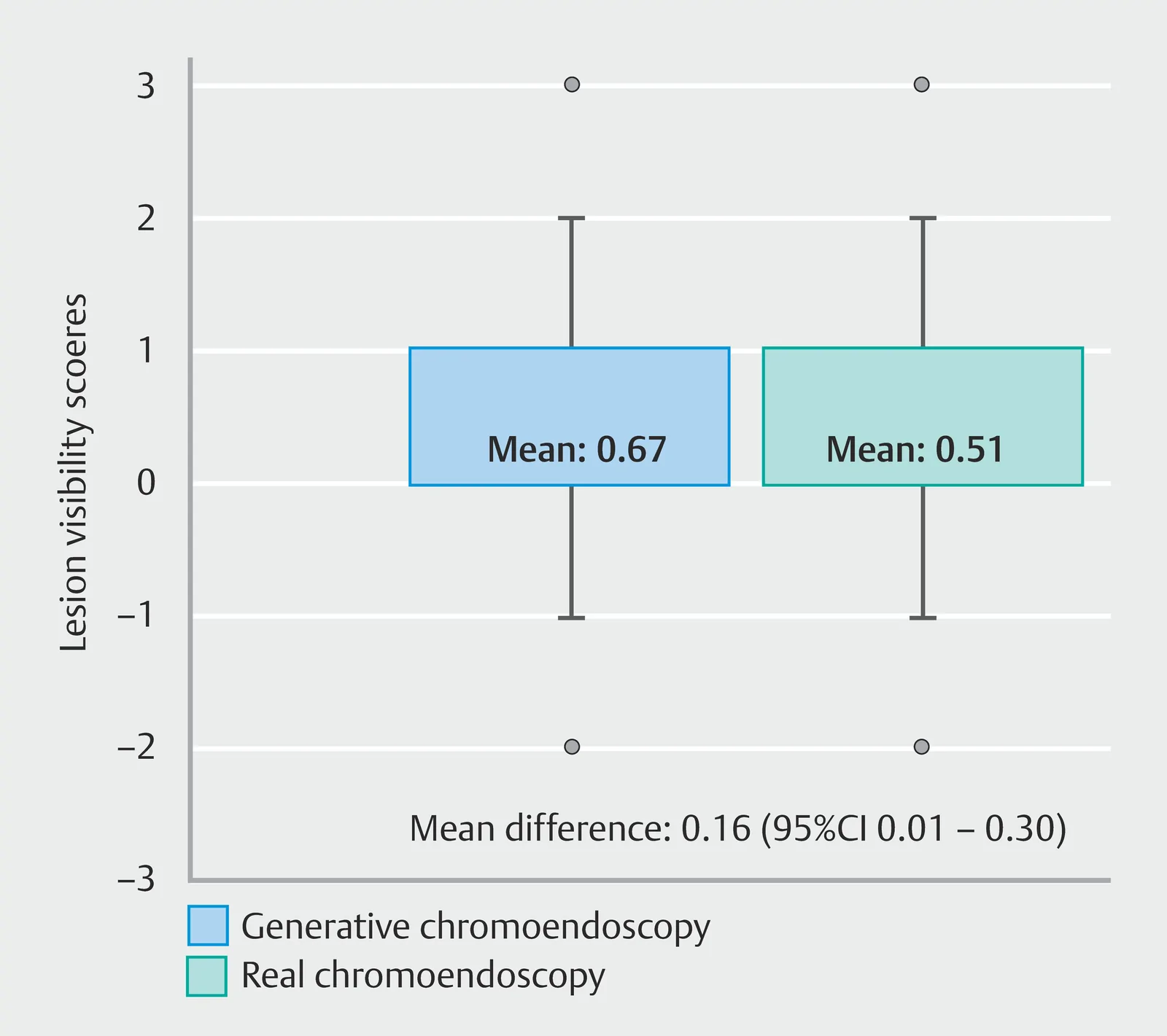
Distribution of lesion visibility scores for generative chromoendoscopy and real chromoendoscopy. Mean (SD) visibility scores were 0.67 (0.93) for generative chromoendoscopy and 0.51 (1.00) for real chromoendoscopy.

Representative endoscopic images obtained with WLI, generative chromoendoscopy, and real chromoendoscopy are shown in Fig. 1 and Video 1 . A total of 360 evaluations were obtained for both generative and real chromoendoscopy (60 lesions, each assessed by an operator and an assistant at three viewing distances). The distribution of lesion visibility scores is presented in Fig. 2 . The mean (SD) visibility scores were 0.67 (0.93) for generative chromoendoscopy, and 0.51 (1.00) for real chromoendoscopy. The mean difference between generative chromoendoscopy and real chromoendoscopy was 0.16 (95%CI 0.01 to 0.30). The ICC for generative chromoendoscopy visibility scores, calculated as facility variance/(facility variance + physician variance + residual variance), was 0.06 (95%CI 0.00 to 0.18).

When stratified by observation distance, the mean (SD) visibility scores for generative chromoendoscopy and real chromoendoscopy were 0.57 (0.92) and 0.46 (0.96; difference 0.11, 95%CI −0.13 to 0.35) at distant view, 0.67 (0.96) and 0.48 (1.02; difference 0.18, 95%CI −0.07 to 0.43) at medium view, and 0.78 (0.90) and 0.60 (1.04; difference 0.18, 95%CI −0.07 to 0.42) at close view.

### Factors affecting gastric lesion visibility with generative chromoendoscopy

The analysis of factors associated with positive lesion visibility using generative chromoendoscopy is summarized in Table 3 . Among 360 assessments for generative chromoendoscopy, 201 (55.8%) yielded positive visibility scores (+1 to +3). Among lesion and background mucosal characteristics, differentiated-type adenocarcinoma (odds ratio [OR] 2.19, 95%CI 1.03 to 4.62) and current *H. pylori* infection (OR 2.14, 95%CI 1.02 to 4.46) were significantly associated with positive visibility scores for generative chromoendoscopy. Endoscopist expertise (board-certified trainers of JGES) was also significantly associated with positive visibility outcomes (OR 2.14, 95%CI 1.22 to 3.76). The distinction between older and newer endoscopy system was not associated with positive visibility outcomes (CV1500/EP8000 vs. CV290/VP7000: OR 1.27, 95%CI 0.56 to 2.91).

**Table:** **Table 3** Univariate analysis of factors associated with positive visibility using generative chromoendoscopy.

Variable	Category (n)	Odds ratio	95%CI	*P* value
*H. pylori* status	Negative/eradicated (288)	Ref		
	Current infection (72)	2.14	1.02 to 4.46	0.04
Lesion morphology	Flat (30)	Ref		
	Elevated (150)	1.66	0.70 to 3.93	0.25
	Depressed (180)	1.94	0.82 to 4.58	0.13
Lesion size	<10 mm (150)	Ref		
	≥10 mm (210)	0.99	0.63 to 1.56	0.96
Histologic type	Undifferentiated (42)	Ref		
	Differentiated (252)	2.19	1.03 to 4.62	0.04
	Others (66)	2.27	0.95 to 5.42	0.06
Invasive depth	Adenoma (48)	Ref		
	pT1a (240)	1.34	0.67 to 2.70	0.41
	pT1b (72)	1.35	0.60 to 3.04	0.46
Mucosal atrophy	C-0 (60)	Ref		
	C1–3 (60)	1.22	0.59 to 2.54	0.58
	O1–3 (240)	1.18	0.61 to 2.31	0.62
Intestinal metaplasia	Absent (96)	Ref		
	Present (264)	1.09	0.64 to 1.84	0.76
Endoscopist level	Nonexpert (198)	Ref		
	Expert (162)	2.14	1.22 to 3.76	0.01
Endoscopy system	CV290/VP7000 (78)	Ref		
	CV1500/EP8000 (282)	1.27	0.56 to 2.91	0.57
Ref, reference.				

## Discussion

To the best of our knowledge, this is the first prospective clinical proof-of-concept study to evaluate the feasibility of a deep learning-based generative chromoendoscopy system in patients with gastric neoplasms. The generative chromoendoscopy system demonstrated improved visibility of preidentified gastric neoplasms compared with standard WLI and showed visibility comparable with that of real chromoendoscopy. Its performance was particularly enhanced for differentiated-type adenocarcinomas, in patients with current *H. pylori* infection, and when assessments were performed by expert endoscopists.

IEE is widely used for the diagnosis of superficial gastrointestinal lesions and can be classified into two main types: classical IEE, which uses dye spraying; and digital IEE, which modifies light wavelengths. Indigo carmine chromoendoscopy is a classical IEE technique that highlights subtle mucosal irregularities and color contrast; however, practical drawbacks – including increased time, labor, and cost associated with dye preparation and spraying – have limited its widespread adoption. Digital IEEs have been developed as alternatives and are widely used; however, their effectiveness for detecting gastric neoplasms remains debated. Among digital IEE modalities, narrow-wavelength techniques such as narrow-band imaging (NBI) are most widely used. NBI has demonstrated superior performance compared with WLI for demarcation of gastric neoplasms and differentiation from non-neoplastic lesions 16 17 . Its superiority over WLI for detection of gastric neoplasms has not however been established 18 . In randomized controlled trials (RCTs), gastric cancer detection rates were 1.9% with WLI versus 2.3% with second-generation NBI, and 5.6% with WLI versus 7.3% with third-generation NBI, with no significant differences observed 19 20 . Therefore, there remains a need for novel observation systems aimed specifically at improving the detection of gastric neoplasms and facilitating early diagnosis, including approaches such as generative chromoendoscopy.

Generative chromoendoscopy based on deep learning has been proposed as a third category of IEE, complementing dye-based and digital modalities. Several retrospective studies have reported its potential utility 8 21 22 23 . In our previous study, whole-lesion area diagnostic performance for gastric neoplasms was slightly higher with generative chromoendoscopy (28%) than with WLI (26%) 8 . Lu et al. 23 also reported similar quality ratings between generative and real chromoendoscopy for surface structure, lesion border, and overall contrast. These analyses were however limited to recorded images. To date, no studies have evaluated the clinical application of generative chromoendoscopy in real time during routine endoscopic procedures.

The present study introduces a generative chromoendoscopy system capable of real-time image conversion and display, demonstrating improved visibility of already recognized gastric neoplasms. One possible explanation for the comparable visibility between generative and real chromoendoscopy is the absence of indigo carmine pooling. The predominant morphology of gastric neoplasms is depressed (e.g. type 0–IIc or 0–III). In real chromoendoscopy, dye may accumulate in depressed areas and obscure surface irregularities, particularly in deeply depressed lesions or when excessive dye is applied. These findings suggest that generative chromoendoscopy may serve as an adjunctive visualization tool for already identified lesions.

At the same time, real-time generative artificial intelligence (AI) introduces potential risks, including distortion, omission, or hallucination of diagnostically relevant features. The generated images do not necessarily represent objective reality, and the underlying generation process is not fully interpretable, making it difficult to provide a definitive medical rationale. Consequently, there is a risk that the system may generate nonexistent findings, produce anatomically inconsistent images, or misidentify artifacts as pathologic features. The system may also highlight non-neoplastic areas, potentially increasing false-positive findings during detection. In addition, we observed occasional distortion of lesion boundaries with generative chromoendoscopy, suggesting that the system may be less suitable for precise delineation. Furthermore, the legal and regulatory framework governing generative AI in clinical practice remains underdeveloped. Clear definitions of responsibility and accountability are needed in cases of AI-related diagnostic errors. To enable safe clinical implementation, it will be essential to validate system performance through rigorous clinical studies and to position the system as a decision-support tool rather than a standalone diagnostic modality.

Analysis of factors associated with generative chromoendoscopy performance provides additional insights. Differentiated-type adenocarcinoma and current *H. pylori* infection are often associated with mucosal irregularities that enhance contrast between lesions and surrounding mucosa, making chromoendoscopy particularly effective. In the present study, the association between improved visibility with generative chromoendoscopy and these factors suggests that the system reproduces key diagnostic features of real chromoendoscopy; however, reduced visibility for undifferentiated and flat-type lesions remains a challenge. As the prevalence of *H. pylori* infection declines, small, flat, and undifferentiated gastric cancers may become more common. Further refinement will be required to improve performance for these lesion types and maintain clinical relevance.

This study has several strengths and limitations. Its primary strength lies in its being the first human study to evaluate generative chromoendoscopy, a novel IEE modality based on machine learning, during live endoscopy. Nevertheless, several limitations should be acknowledged. First, visibility assessment remained inherently subjective. The Hawthorne effect, whereby endoscopists may alter their behavior when aware of being observed, may have contributed to higher visibility scores for generative chromoendoscopy. Ideally, additional parameters – such as consistency with WLI images, preservation of lesion margins, and evaluation of potential artifacts or hallucinations – should also be assessed. Future studies will incorporate these parameters to enable a more comprehensive evaluation using high quality endoscopic video data or live procedures.

Second, the analysis was limited to patients with confirmed gastric neoplasms, meaning the system’s potential to misclassify non-neoplastic mucosa as neoplastic, potentially increasing false-positive rates, could not be evaluated. We were also unable to assess scenarios in which subtle, previously unrecognized neoplasia was present. Consequently, detection performance in real-world clinical settings remains unknown. The ultimate goal of this system is to improve detection of gastric neoplasms in patients undergoing screening or surveillance esophagogastroduodenoscopy. Future studies are planned to evaluate detection performance in such settings, including identification of lesions not initially recognized by endoscopists. RCTs will be required to objectively assess the detection performance of generative chromoendoscopy.

In conclusion, this prospective multicenter proof-of-concept study demonstrated that deep learning-based generative chromoendoscopy is feasible during live endoscopic procedures. Under controlled conditions involving preidentified lesions, it improved the visibility of gastric neoplasms compared with WLI and achieved visibility comparable to real chromoendoscopy, particularly for differentiated adenocarcinomas in patients with current *H. pylori* infection.

## Data transparency statement

Deidentified datasets and the study protocol are available upon reasonable request through the UMIN Individual Case Data Sharing System ( https://www.umin.ac.jp/icds/index.html ).
